# Melatonin ameliorates zearalenone-induced ovarian damage in mice through antioxidative effects

**DOI:** 10.3389/fvets.2025.1587391

**Published:** 2025-08-11

**Authors:** Yi Song, Zhong Guo, Lei Song, Jianxiu Ma, Zhifang Zhao, Yanqing Ma, Xiaoyue Ma, Wenjie Jiang, Wanjing Wang, Chongran Liu, Tongtong Wei, Ling Fu, Zhengli Qi, Jin Zhao

**Affiliations:** ^1^College of Veterinary Medicine, Gansu Agricultural University, Lanzhou, China; ^2^Medical College of Northwest Minzu University, Lanzhou, China; ^3^Key Laboratory of Environmental Ecology and Population Health in Northwest Minority Areas, Medical College of Northwest Minzu University, Lanzhou, China

**Keywords:** melatonin, zearalenone, ovarian, ATM-Chk2-P53 signaling pathway, γH2AX, oxidative stress

## Abstract

**Introduction:**

Zearalenone (ZEN), a mycotoxin from *Fusarium* species, is widely present in contaminated grains and animal products. It exerts estrogen-like effects, disrupting hormonal balance and reproductive function, partly through oxidative stress-induced DNA damage. The ATM-Chk2-p53 pathway is a key mediator of the DNA damage response. Melatonin (MT), a natural antioxidant, supports ovarian function by regulating hormone secretion and reducing oxidative stress. This study explores whether MT alleviates ZEN-induced ovarian and granulosa cell damage via the ATM-Chk2-p53 pathway.

**Methods:**

Female mice were exposed to ZEN (0.8mg/kg) with or without MT (10, 20, or 40 mg/kg) for 28 days. Ovarian morphology, hormone levels, oxidative stress markers, and DNA damage proteins were assessed. GRM02 cells were treated with ZEN (60 μM) and/or MT (100 μM). Apoptosis, cell cycle, oxidative stress, and DNA damage markers were evaluated. ATM-knockout and ATM-activated GRM02 models were used to examine pathway involvement.

**Results:**

ZEN caused ovarian atrophy, estrous disruption, reduced E2, FSH, and LH, elevated oxidative stress, and increased γH2AX, pATM, Chk2, and p53 expression. MT restored ovarian function, improved antioxidant capacity, and reduced DNA damage. In GRM02 cells, MT mitigated ZEN-induced G2/M arrest, apoptosis, and oxidative stress. ATM activation enhanced MT’s protective effect, while ATM knockout worsened ZEN toxicity.

**Discussion:**

MT protects against ZEN-induced ovarian and cellular damage by reducing oxidative stress and modulating the ATM-Chk2-p53 pathway. These findings highlight MT’s potential as a protective feed additive against mycotoxin-related reproductive toxicity.

## 1 Introduction

Zearalenone (ZEN) is a mycotoxin with estrogenic activity produced by *Fusarium* species (1). It predominantly originates from contamination during the cultivation phase of grain crops and is closely associated with inadequate management practices during storage and transportation. High doses of ZEN (1.5 mg /kg) can cause reproductive disorders in female animals, such as delayed estrus, estrus disturbance, vaginal inflammation, swollen vulva, pseudo-pregnancy, infertility, stillbirths, and miscarriages, severely affecting reproductive health (2, 3). Given this, it is significant to explore new and effective ways to alleviate the toxicity of ZEN. ZEN causes oxidative stress by generating reactive oxygen species (ROS), leading to cellular damage like lipid peroxidation, protein oxidation, and DNA damage, which can result in cell death. Studies indicate that ZEN exposure significantly raises ROS levels, worsening oxidative stress and harming reproductive and other organs (4, 5). ZEN also disrupts mitochondrial function, crucial for energy balance and apoptosis regulation, by impairing the electron transport chain. This increases ROS production and mitochondrial dysfunction, triggering apoptosis and autophagy, and furthering cellular damage and toxicity (6, 7). ZEN affects oxidative stress and influences antioxidant response pathways, particularly the PI3K/Akt/Nrf2 pathway, which boosts antioxidant enzyme expression for cellular protection (4, 7).

Oxidative stress can lead to DNA damage, known as the DNA damage response (DDR), or replication stress, with double-strand breaks (DSBs) representing the most severe form of damage (8). The ataxia-telangiectasia mutated (ATM) protein, a serine/threonine protein kinase, serves as a pivotal regulator of the DDR. Upon the occurrence of DSBs, the ATM-Chk2-p53-dependent DDR pathway is activated, facilitating DNA repair, cell cycle arrest, apoptosis, or senescence (9, 10). Beyond its essential role in the repair of DNA double-strand breaks, ATM is also instrumental in responding to oxidative stress and maintaining intracellular redox homeostasis. These functions underscore the significance of ATM as a critical target for research into the DNA damage response and oxidative stress response (11, 12).

In mammalian ovaries, ovarian granulosa cells (GC) are integral to the growth and maturation of ovarian follicles, as they secrete a variety of factors that facilitate oocyte development, proliferation, and differentiation (13). The apoptosis of granulosa cells can result in follicular atresia (14). ZEN induces apoptosis in ovarian granulosa cells, impedes oocyte maturation, and elevates intracellular ROS levels, thereby causing damage to ovarian tissues and resulting in lesions within the rat reproductive system (15, 16). Consequently, ovarian GC may serve as a promising target for research on reproductive function in females affected by ZEN exposure.

Melatonin (MT), an indoleamine hormone predominantly secreted by the pineal gland within the hypothalamus of mammals, has been extensively studied for its involvement in follicular development (17, 18). It has been demonstrated that melatonin present in human follicular fluid mitigates oxidative stress, thereby safeguarding oocytes and granulosa cells. Furthermore, melatonin receptors have been identified in ovarian granulosa cells, where they contribute to the regulation of the ovarian circadian rhythm (19, 20). During the process of follicular development, melatonin concentrations are markedly elevated in large follicles compared to small ones, and pre-ovulatory follicular melatonin levels exceed those found in serum. These observations underscore the pivotal role of melatonin in follicular maturation and ovulation (21, 22). Moreover, melatonin functions as an endogenous antioxidant, with its derivatives demonstrating significant direct free radical scavenging capabilities. The antioxidant and stress-protective properties of melatonin in human and mouse oocytes effectively neutralize ROS within these cells, thereby mitigating oxidative stress-induced damage during oocyte maturation (23). In addition, melatonin enhances the antioxidant capacity of oocytes and granulosa cells by upregulating the expression of antioxidant enzymes, such as superoxide dismutase (SOD) and glutathione peroxidase (GPx) (24). Due to its straightforward synthesis, high biosafety profile, and ease of accumulation, melatonin presents significant clinical application potential and extensive developmental prospects.

This study aims to examine the deleterious effects of ZEN on murine ovarian tissue and ovarian granulosa cells (GRM02), and to investigate the potential of MT in mitigating oxidative stress induced by ZEN in ovarian tissue and GRM02 cells, thereby enhancing ovarian tissue function and reducing oxidative damage in GRM02 cells. By integrating oxidative stress and DNA damage pathways, this research explores the role of the ATM-Chk2-p53 signaling pathway in the ameliorative effects of MT on ZEN-induced oxidative damage in GRM02 cells. The anticipated findings are expected to provide a deeper understanding of the mechanisms underlying ZEN’s reproductive toxicity and offer theoretical support for the use of MT as a protective feed additive.

## 2 Materials and methods

### 2.1 Animals

The Laboratory Animal Ethics Committee of Northwest Minzu University approved all animal experiments. We followed ARRIVE guidelines and the Ministry of Science and Technology’s Experimental Animal Guidelines of China. We used 120 female Kunming mice, aged 6 weeks and weighing 28–30 g, sourced from the Lanzhou Veterinary Research Institute, Chinese Academy of Agricultural Sciences (Approval No. SCXK (Gansu) 2020–0002). All mice were kept in standard conditions (23 ± 2°C, 45–55% humidity, 12-h light cycle) with food and water. They were humanely sacrificed at the study’s end. Female 5-week-old Kunming mice (n = 120) were monitored for 1 week to confirm their estrous cycle. The mice were then divided into six groups of 20 each: Group 1: control (0.2 mL saline, ip), Group 2: MT (20 mg/kg, ip), Group 3: ZEN (0.8 mg/kg, i.g.), Group 4: ZEN (0.8 mg/kg, i.g.) + MT (10 mg/kg, ip), Group 5: ZEN (0.8 mg/kg, i.g.) + MT (20 mg/kg, ip), Group 6: ZEN (0.8 mg/kg, i.g.) + MT (40 mg/kg, ip). Drugs were administered daily for 28 days.

The dosage and route of administration for melatonin were determined in accordance with existing literature (25). Similarly, the dosage and administration route for ZEN were informed by prior research, with a specified administration dosage of 0.8 mg/kg, i.g. Melatonin was given in the afternoon, 16 h before the next sunrise, to extend daily exposure until natural nocturnal secretion occurred (26).

### 2.2 Evaluation of ovarian function

#### 2.2.1 Determination of estrous cycle

The mice were manually restrained, and their calvaria exposed. Vaginal lavage fluid was collected with 20 μL of saline, spread on slides, and immersed in a solution of 20% formaldehyde, 95% alcohol, and 5% glacial acetic acid for 10 min. The slides were then stained with hematoxylin and eosin (H&E) to determine the estrous cycle stages under a microscope. The estrus cycle stages were identified by the dominant cell type in vaginal smears (27). Vaginal lavage fluid was collected daily from 7:30 to 8:30 am for 15 days.

#### 2.2.2 Counts and morphological analysis of ovarian follicles

Ovaries were fixed in 4% paraformaldehyde for a week, embedded in paraffin, sectioned at 6 μm, mounted on slides, and stained with PAS. Every fifth section of each ovary was microscopically examined, including only follicles with a visible nucleus to avoid duplication. Follicles were classified based on established criteria (28).

#### 2.2.3 Enzyme-linked immunosorbent assay (ELISA) of E2, LH and FSH

Blood samples were collected post-experiment, clotted overnight at 4°C, and centrifuged twice at 3500 rpm for 20 min. The serum was analyzed for FSH, LH, and E2 using spectrophotometry with ELISA kits (Mlbio, China), and standard protein curves were calculated.

### 2.3 Immunohistochemistry

After a 4-week treatment, the mice were euthanized, and their ovaries were fixed in 4% paraformaldehyde. Paraffin-embedded sections were then dewaxed, rehydrated, and underwent antigen retrieval using citrate buffer (pH 6). The sections were incubated overnight at 4°C with primary antibodies against anti-phospho-H2AX Ser 139 (γH2AX; 05–636; dilution, 1:500; Millipore), anti-phospho-ATM (Ser1981; 05–740, dilution, 1:300; Millipore), p53 (AF0879; dilution, 1:300; Affinity Bioscience, United States) and Chk2 (ab199031; dilution, 1:500; Abcam, United States). In the negative control experiment, phosphate-buffered saline (PBS) replaced the primary antibody. The sections were then treated with a horseradish peroxidase-conjugated secondary antibody (SP-9001; ZSGB-BIO ORIGEN) and incubated for 20 min at room temperature. Cell visualization was done using 3,3-Diaminobenzidine (ZLI-9018; ZSGB-BIO ORIGENE), with hematoxylin for nuclear counterstaining. All sections were incubated under consistent conditions, including antibody concentrations.

### 2.4 Oxidative stress status analysis

The serum and cell antioxidant indexes were assessed utilizing commercial kits obtained from the Jiancheng Bioengineering Institute in Nanjing, China. The total antioxidant capacity (T-AOC), the total superoxide dismutase (SOD), glutathione peroxidase (GSH-Px), catalase (CAT), and malondialdehyde (MDA) levels were determined following the manufacturer’s guidelines. Absorbance readings for these analyses were recorded using a microplate reader from Tecan in Austria.

### 2.5 Cell lines

The mouse ovarian granulosa cells (GRM02) were obtained from the Shanghai Institute of Biochemistry and Cell Biology in Shanghai, China. The cells were maintained in DMEM/F12 (1:1) supplemented with 10% fetal bovine serum (FBS) at 37°C in a 5% CO_2_ atmosphere. GRM02 cells were exposed to a 10 μM concentration of the ATM Activator Chloroquine diphosphate (Selleckchem, Houston, TX, United States) for a duration of 2 h in order to induce ATM activation for subsequent experimental procedures.

### 2.6 Knockout of ATM by guide RNA (gRNA) /Cas9

Initially, the target sequences for ATM knockdown were identified. Given that the coding sequences of ATM-201 encompass approximately 18 kb, a strategy involving the knockdown of the majority of exons and coding sequences was deemed appropriate. This approach can be effectively implemented by designing a double gRNA/Cas9 vector to achieve the desired ATM knockdown. Specifically, exon 4 was selected for the design of upstream and downstream gRNAs. The gRNA sequences were designed using the online tool available at http://crispor.tefor.net/, and the resulting gRNA sequences are presented in Table 1. The px459-puro vector was utilized as the backbone for constructing the dual gRNA/Cas9 expression vector. Consistent with the aforementioned GRM02 mouse ovarian granulosa cell liposome transfection protocol, GRM02 mouse ovarian granulosa cells in optimal condition were seeded into a 12-well plate. Upon reaching approximately 80% confluence, a transfection solution was prepared by mixing 2 μg/mL of plasmid DNA with 2 μL of liposome per well, totaling 100 μL per well. This mixture was incubated at room temperature for 20 min before being gradually added to the GRM02 mouse ovarian granulosa cells. The px459-EF1a-dualH ATM18exon-puro recombinant vector was transfected into three replicate wells, with one untransfected well serving as a negative control for screening purposes. The ATM knockout cell line of GRM02 mouse ovarian granulosa cells was established 48 h post-transfection. Monoclonal cell isolation was conducted in three separate batches. During each passage, a small number of cells were lysed and subjected to PCR amplification and sequencing for verification.

**Table 1:** Design of gRNA sequences for the ATM gene.

Position	Name	Sequence	PAM
Knockout	ATM-sgRNA1	GATAGTGACAAACCTAGGCCA	GGG
	ATM-sgRNA2	GAGGACACTTCCTGACAGTGA	TGG

### 2.7 Cell viability assay

The MTT assay was used to detect the effects on ZEN-treated/ MT-treated cell viability. GRM02 cells were cultured in 96-well plates. When reaching a density of 1 × 10^4^ cells per well,cells were treated with different concentrations of ZEN (0, 15, 30, 60, and 120 μM) or MT (0, 1, 10, 100, 1,000 μM) for 24 h (29). MTT working solution (5 mg/mL in PBS) was added to each well and incubated at 37°C for 3 h. The optical density (OD) was then measured at 490 nm using a microplate reader (Sunrise, TECAN, Männedorf, Switzerland). Cell viability was determined by calculating the percentage of viable cells in the melatonin or ZEN-treated group compared to the untreated control using the formula: cell viability (%) = [OD (Treated) − OD (blank)]/[OD(Control) − OD (Blank)] × 100.

### 2.8 Cell cycle analysis

The cell cycle was analyzed using a cell cycle kit. Initially, 5 × 10^5^ GRM02 cells were treated with optimal concentrations of ZEN for 24 h, followed by optimal concentrations of MT for another 24 h. Then, 1 × 10^6^ treated cells were collected, rinsed with ice-cold PBS, fixed, and permeabilized with 70% ethanol in PBS at −20°C for at least 1 h. After fixation, the cells were washed with PBS and stained with propidium iodide/RNase buffer at 4°C in the dark. at least 15 min. Stained cells were promptly subjected to analysis for propidium iodide fluorescence using the BD FACSCanto II.

### 2.9 Cell apoptosis assay

The apoptotic rate of treated GRM02 cells was measured using the Annexin V-fluorescein isothiocyanate (FITC)/propidium iodide (PI) apoptosis detection kit (Liankebio, Hangzhou, China). The cells were harvested, incubated with 100 μL of Annexin V-FITC/PI reagent for 20 min in the dark to label phosphatidylserine and DNA content. The apoptotic GRM02 cells were divided into quadrants based on fluorescence: Q4 for healthy cells (FITC-/PI-), Q3 for early apoptotic cells (FITC+/PI-), and Q2 for necrotic and late apoptotic cells (FITC+/PI+). The apoptosis rate was calculated as: (Early apoptotic cells + advanced apoptotic cells) / total cell number × 100%.

### 2.10 Western blot assay

Total protein samples from treated GRM02 cells were extracted using cell lysis buffer (Abcam; Cat. no. ab152163) supplemented with protease inhibitors (Roche, Basel, Switzerland; Cat. no. 04693132001). Equal amounts of protein samples were separated using the Bio-Rad Bis-Tris Gel system and subsequently transferred to polyvinylidene fluoride (PVDF) membranes (Millipore, Billerica, MA, United States; Cat. no. IPVH00010). Primary antibodies, including anti-phospho-H2AX Ser 139 (γH2AX; 05–636; dilution, 1:2000; Millipore), anti-phospho-ATM(Ser1981; 05–740; dilution, 1:1000; Millipore), p53 (AF0879; dilution, 1:800; Affinity Bioscience, United States) and Chk2 (ab199031; dilution, 1:1000; Abcam, United States), and anti-GAPDH (ab2701826; dilution, 1:5000; Abcam, United States), were incubated with PVDF membranes at 4°C overnight, followed by incubation with a secondary antibody (ab6721; dilution, 1:5000; Abcam, United States). Protein band intensities were visualized using the enhanced chemiluminescent visualization (ECL) system (Pierce, Rockford, IL, United States; Cat. no. 32106) after washing.

### 2.11 Statistical analysis

Each experiment in the study was repeated a minimum of three times independently. The findings were reported as the mean ± standard error of the mean and were analyzed utilizing SPSS 26.0 software (IBM, United States) and GraphPad Prism 9 (GraphPad Software, San Diego, CA, United States). The normality of the data was evaluated through the Shapiro–Wilk test, while Bartlett’s test was employed to assess the homogeneity of variance. Statistical significance between experimental groups was determined using a one-way ANOVA, followed by *post-hoc* comparisons utilizing Tukey’s multiple comparisons test. Bivariate correlation analyses were conducted using Pearson’s correlation coefficient. Statistical significance is indicated by the *p* value, *p < 0.05*, marked with different characters.

## 3 Results

### 3.1 Melatonin ameliorates ZEN-induced ovarian dysfunction

**Figure 1:**
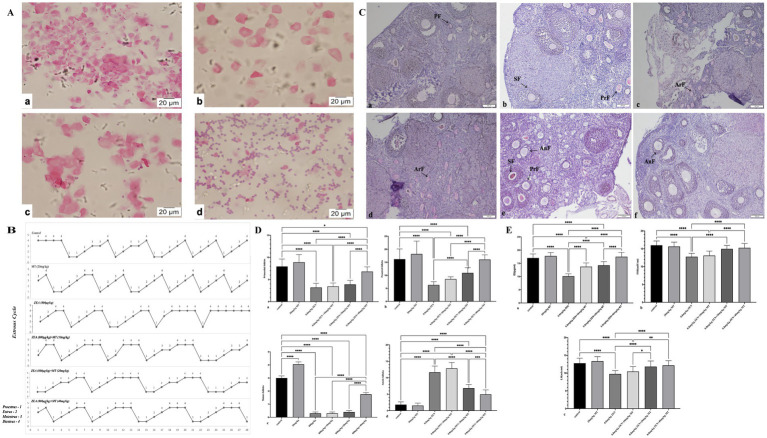
Melatonin can improve the damage to ovarian reproductive function exposed to ZEN. Panel **(A)** shows microscopic images of cellular samples, stained in pink, at high magnification with scale bars indicating 20 micrometers. **(a)** Proestrus (H&E, 40X); **(b)** Estrus (H&E, 40X); **(c)** Metestrus (H&E, 40X); d Diestrus (H&E, 40X). Panel **(B)** includes line graphs displaying estrous cycle variations across different conditions. Proestrus-1; Eestrus-2; Metestrus-3; Diestrus-4. Panel **(C)** presents histological sections of tissue labeled with annotations such as PF, SF, PrF, ArF, and AnF. **(a)** Control group. **(b)** MT(20mg/kg) group. **(c)** ZEN(0.8mg/kg) group. **(d)** 0.8mg/kg(ZEN)+10mg/kg(MT) group. **(e)** 0.8mg/kg(ZEN)+20mg/kg(MT) group. **(f)** 0.8mg/kg(ZEN)+40mg/kg(MT) group. (PAS, 10X). Panels **(D,E)** feature bar graphs comparing various measurements with statistical significance denoted by asterisks. Each graph compares control groups with experimental groups, focusing on reproductive biology data.

The estrous cycle in mice generally spans 4 to 5 days and encompasses four distinct phases: proestrus, estrus, metestrus, and diestrus, which can be identified through vaginal smear analysis (Figure 1A). The findings demonstrated that both the control group and the group treated with melatonin (MT) at a dosage of 20 mg/kg maintained regular estrous cycles, with MT administration mitigating stress-induced disruptions in the cycle during the initial phase of the study. Conversely, the group administered with zearalenone (ZEN) at 0.8 mg/kg exhibited irregular estrous cycles, notably characterized by the absence of the estrus phase. The groups receiving combined treatments of ZEN (0.8 mg/kg) with MT at dosages of 10 mg/kg and 20 mg/kg showed partial recovery, as evidenced by the presence of the estrus phase; however, the cycle duration extended to 6 to 8 days. Importantly, the group treated with ZEN (0.8 mg/kg) and MT at a dosage of 40 mg/kg demonstrated the most pronounced protective effect, with the estrous cycle duration reverting to the normal range of 4 to 5 days (Figure 1B).

After completing the experiment, the ovaries were meticulously dissected, and their dimensions and weights are detailed in Table 2. The ovarian weight in the ZEN (0.8 mg/kg) group was significantly greater than that in the MT (20 mg/kg) group, with statistical significance. No significant differences in ovarian weight were detected among the various MT treatment groups. In terms of ovarian diameter, both the ZEN and ZEN + MT (10 mg/kg) groups exhibited significantly larger diameters compared to the control group. Conversely, the ZEN + MT (20 mg/kg) and ZEN + MT (40 mg/kg) groups demonstrated a significant reduction in diameter. However, no statistically significant differences in ovarian diameter were observed between the treatment groups.

**Table 2:** Change in ovaries size and body weight after administration of ZEN and MT.

Group	Ovarian sizes (mm)	Ovarian weights (mg)
Control	3.11 ± 0.07^ac^	8.81 ± 0.50^ab^
MT (20 mg/kg)	3.23 ± 0.02^ac^	8.49 ± 0.33^b^
ZEN (800 μg/kg)	3.71 ± 0.05^b^	10.78 ± 0.43^a^
ZEN (800 μg/kg) + MT (10 mg/kg)	3.51 ± 0.09^bc^	9.98 ± 0.69^ab^
ZEN (800 μg/kg) + MT (20 mg/kg)	3.35 ± 0.08^ac^	9.36 ± 0.58^ab^
ZEN (800 μg/kg) + MT (40 mg/kg)	3.44 ± 0.08^ac^	9.35 ± 0.45^ab^

Change in ovarian size and body weight after administration of ZEN and MT. The same letters mean no significant difference, the different letters (a, b, c) mean a significant difference at *p <* 0.05.

Periodic acid-Schiff (PAS) staining and light microscopy were employed to evaluate ovarian morphology. In both the control and melatonin (MT, 20 mg/kg) groups, granulosa cells exhibited well-organized structures, and the antral follicles contained substantial fluid. Conversely, in the ZEN and ZEN + MT (10 mg/kg) groups, mature follicles were absent, and the granulosa cells of primordial follicles were arranged in a loose and disordered manner. There was a reduction in the number of primordial follicles, alongside an increase in atretic follicles, which was accompanied by significant ovarian stromal fibrosis. In the ZEN + MT (20 mg/kg) group, although mature follicles were not observed, there was an increase in the number of primary follicles, while the number of atretic follicles remained elevated. In contrast, the ZEN + MT (40 mg/kg) group demonstrated a significant increase in the number of mature and primary follicles, characterized by organized granulosa cell arrangement and a marked reduction in atretic follicles (Figure 1C).

Quantification of primordial, primary, mature, and atretic follicles was performed (Table 3). Compared to the control group, the ZEN (0.8 mg/kg) group exhibited a significant decrease in mature follicles (0.30 ± 0.11), primary follicles (6.30 ± 0.28), and primordial follicles (3.20 ± 0.21; *p* < 0.05), with a significant increase in atretic follicles (11.65 ± 0.42; *p* < 0.05). In comparison, the ZEN (0.8 mg/kg) + MT (40 mg/kg) group demonstrated a significant increase in mature follicles (1.75 ± 0.12), primary follicles (16.05 ± 0.40), and primordial follicles (6.80 ± 0.24; *p* < 0.05), accompanied by a significant reduction in atretic follicles (4.95 ± 0.30; *p* < 0.05). Compared to the ZEN (0.8 mg/kg) + MT (40 mg/kg) group, the ZEN (0.8 mg/kg) + MT (10 mg/kg) and ZEN (0.8 mg/kg) + MT (20 mg/kg) groups exhibited a significant reduction in the numbers of mature, primary, and primordial follicles (*p* < 0.05), while the number of atretic follicles increased (p < 0.05). However, there were no significant differences in follicle numbers between the ZEN (0.8 mg/kg) + MT (40 mg/kg) group and the control group (*p* > 0.05; Figure 1D).

**Table 3:** Mean number of primordial follicles, preantral follicles, mature follicles, and atretic follicles in each group of mice.

Group	Primordial follicles	Preantral follicles	Mature follicles	Atretic follicles
Control	7.95 ± 0.37^a^	16.20 ± 0.87^a^	3.00 ± 0.16^a^	1.85 ± 0.18^a^
MT (20 mg/kg)	8.90 ± 0.39^ab^	18.15 ± 1.11^ab^	4.05 ± 0.18^b^	1.55 ± 0.17^ab^
ZEN (800 μg/kg)	3.20 ± 0.21^c^	6.30 ± 0.28^c^	0.30 ± 0.11^c^	11.65 ± 0.42^c^
ZEN (800 μg/kg) + MT (10 mg/kg)	3.45 ± 0.17^cd^	8.55 ± 0.20^cd^	0.30 ± 0.11^cd^	12.85 ± 0.40^cd^
ZEN (800 μg/kg) + MT (20 mg/kg)	3.90 ± 0.20^cd^	10.90 ± 0.46^d^	0.40 ± 0.11^cd^	6.85 ± 0.24^e^
ZEN (800 μg/kg) + MT (40 mg/kg)	6.80 ± 0.24^e^	16.05 ± 0.40^ab^	1.75 ± 0.12^e^	4.95 ± 0.30^f^

Mean number of Primordial follicles, Preantral follicles, Mature follicles, and Atretic follicles in each group of mice. The same letters mean no significant difference, the different letters (a, b, c, d, e, f) mean a significant difference at *p* < 0.05.

ELISA assays were performed to measure serum levels of E2, FSH, and LH in ovaries from mice treated with ZEN and MT. The results indicated that, compared to the control group, serum levels of E2, FSH, and LH were significantly reduced in the ZEN (0.8 mg/kg) and ZEN (0.8 mg/kg) + MT (10 mg/kg) groups (*p* < 0.05). Conversely, serum levels of E2, FSH, and LH were significantly elevated in the ZEN (0.8 mg/kg) + MT (20 mg/kg) and ZEN (0.8 mg/kg) + MT (40 mg/kg) groups (*p* < 0.05). Compared to the ZEN (0.8 mg/kg) + MT (40 mg/kg) group, the ZEN (0.8 mg/kg) + MT (10 mg/kg) group showed significantly lower levels of E2, FSH, and LH (*p* < 0.05), while the ZEN (0.8 mg/kg) + MT (20 mg/kg) group exhibited a significant decrease in E2 and FSH levels (*p* < 0.05), with no significant change in LH levels (*p* > 0.05; Figure 1E).

### 3.2 Melatonin alleviates ZEN-induced oxidative damage in ovaries

**Figure 2:**
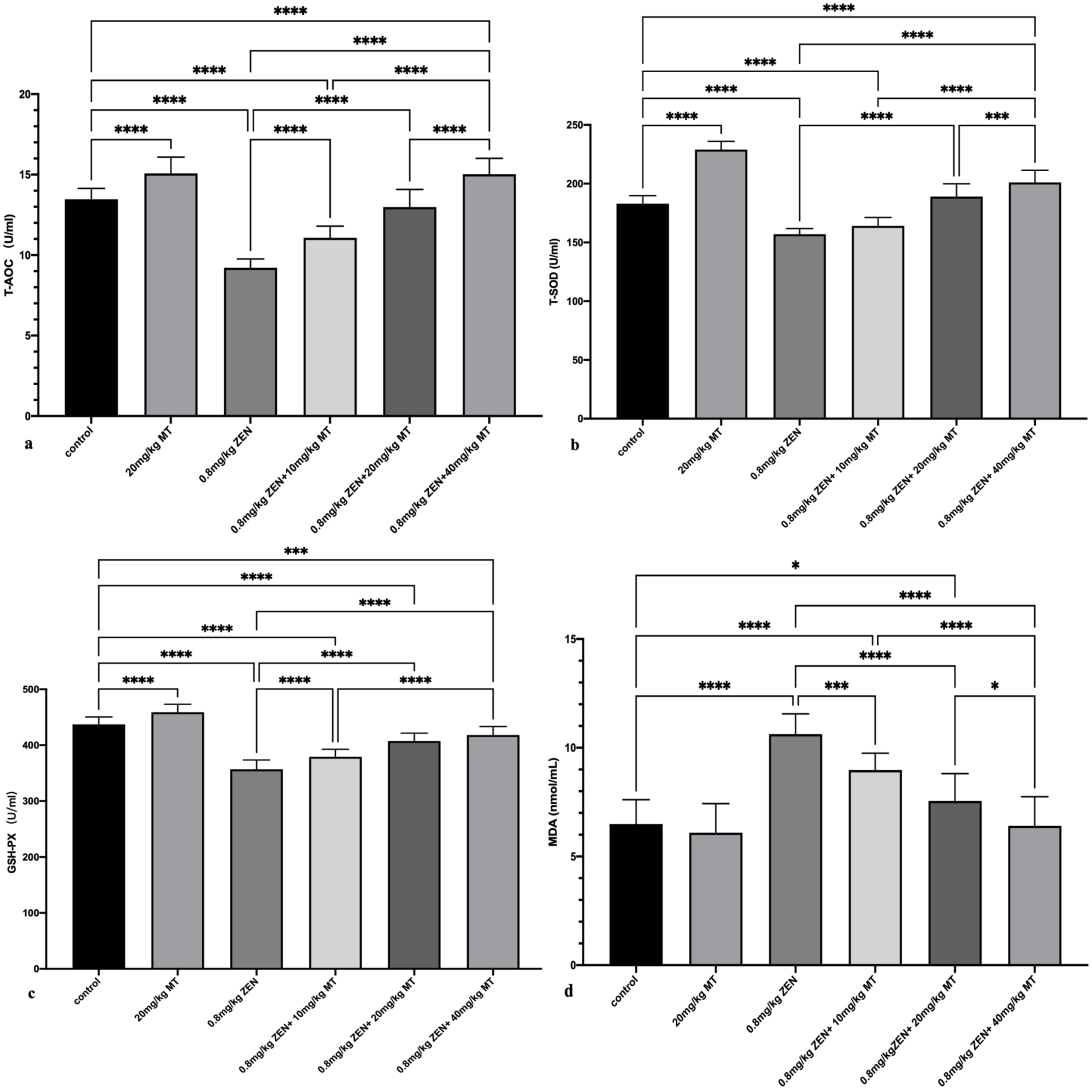
Bar charts **(a-d)** showcasing the effects of different treatments on various biochemical parameters: TAOC, T-SOD, GSH-PX, and MDA. Statistical significance is denoted with asterisks: * *p* < 0.05, ** *p* < 0.01, *** *p* < 0.001, **** *p* < 0.0001. Treatments include a control, MT and ZEN variations.

The results of oxidative stress marker analysis revealed that compared to the control group, both the ZEN (0.8 mg/kg) and ZEN (0.8 mg/kg) + MT (10 mg/kg) groups exhibited significantly reduced levels of antioxidant markers (T-AOC, T-SOD, GSH-PX; *p* < 0.05) and significantly elevated MDA levels (*p* < 0.05). Compared to the ZEN (0.8 mg/kg) group, the ZEN (0.8 mg/kg) + MT (10 mg/kg), ZEN (0.8 mg/kg) + MT (20 mg/kg), and ZEN (0.8 mg/kg) + MT (40 mg/kg) groups showed marked increases in T-AOC, T-SOD, and GSH-PX expression (*p* < 0.05) and significant reductions in MDA levels (*p* < 0.05). Additionally, when compared to the ZEN (0.8 mg/kg) + MT (40 mg/kg) group, the ZEN (0.8 mg/kg) + MT (10 mg/kg) and ZEN (0.8 mg/kg) + MT (20 mg/kg) groups exhibited significantly lower T-AOC, T-SOD, and GSH-PX expression (*p* < 0.05) and higher MDA expression (*p* < 0.05). Moreover, the MT (20 mg/kg) group showed significant increases in T-AOC, T-SOD, and GSH-PX compared to the control group (*p* < 0.05), but no significant change in MDA levels (*p* > 0.05; Figure 2).

### 3.3 Melatonin mitigates ZEN-induced oxidative DNA damage in ovaries

**Figure 3:**
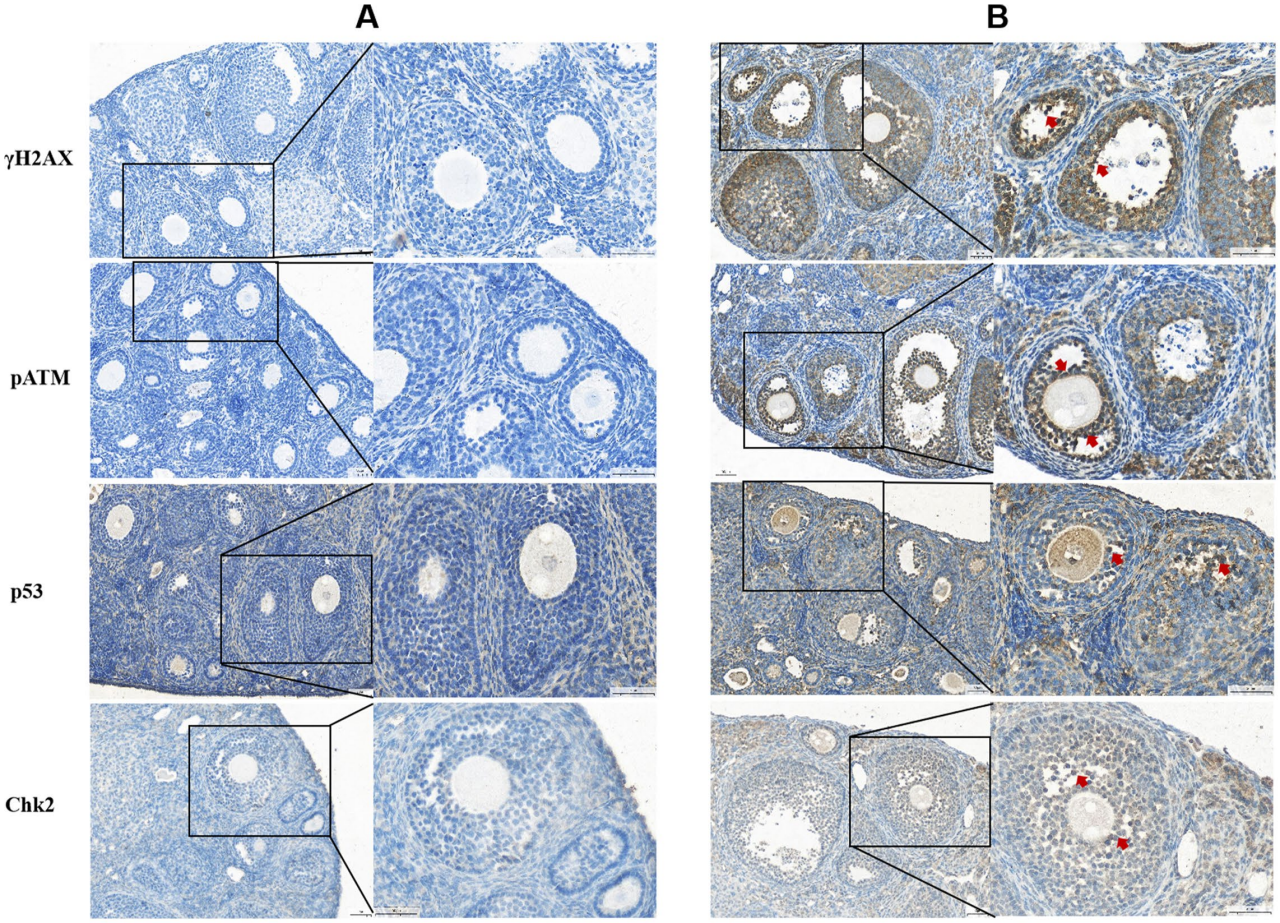
Histological sections labeled **(A)** and **(B)** display cellular staining for γH2AX, pATM, p53, and Chk2. Panel **(A)** shows blue staining with minimal dark regions, indicating baseline levels. Panel **(B)** reveals darker, brown-stained areas marked with red arrows, indicating increased protein expression. Each section has highlighted areas, with magnified insets showing cellular details.

**Figure 4:**
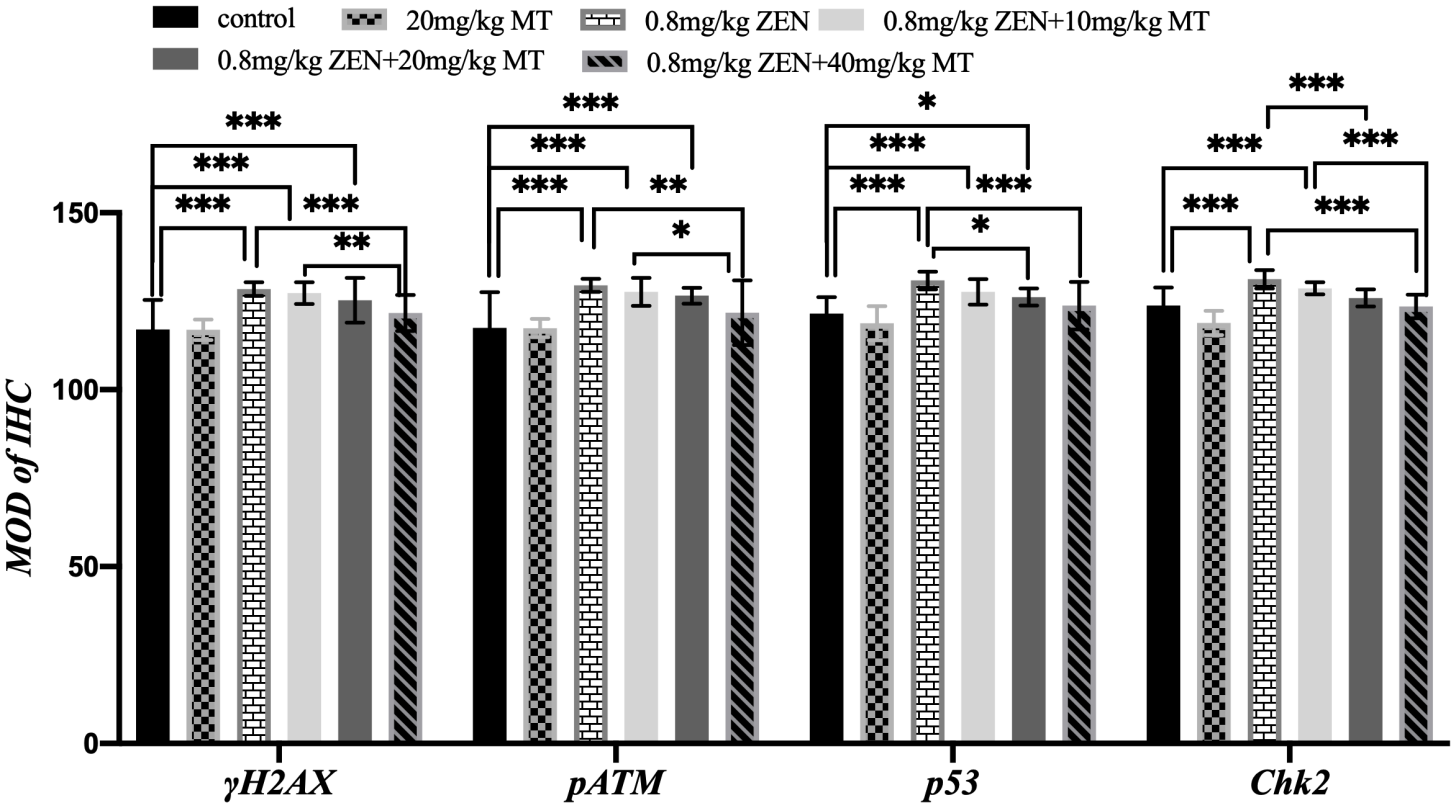
Bar graph comparing the mean optical density (MOD) of immunohistochemistry (IHC) for γH2AX, pATM, p53, and Chk2 across different treatments. Treatments include control, 20 mg/kg MT, 0.8 mg/kg ZEN, 0.8 mg/kg ZEN plus varying doses of MT. Significant differences are indicated with asterisks, with three indicating a high level of significance.

Immunohistochemical analysis revealed that γH2AX, pATM, Chk2, and p53 proteins were predominantly expressed in the granulosa cells (GCs) and oocytes of ovaries from ZEN (0.8 mg/kg)-treated mice, with positive nuclear staining observed (Figure 3). Compared to the control group, the ZEN (0.8 mg/kg) and ZEN (0.8 mg/kg) + MT (10 mg/kg) groups exhibited significantly elevated levels of γH2AX, pATM, Chk2, and p53 expression (*p* < 0.05). In contrast, the ZEN (0.8 mg/kg) + MT (40 mg/kg) group showed significant reductions in all four proteins compared to the ZEN group (*p* < 0.05). The ZEN (0.8 mg/kg) + MT (20 mg/kg) group displayed significant decreases in Chk2 and p53 levels (*p* < 0.05), but the reductions in γH2AX and pATM were not significant (*p* > 0.05). When compared to the ZEN (0.8 mg/kg) + MT (40 mg/kg) group, the ZEN (0.8 mg/kg) + MT (10 mg/kg) group had significantly higher expression levels of γH2AX, pATM, and Chk2 (*p* < 0.05), while p53 expression was higher but did not reach statistical significance (*p* > 0.05; Figure 4).

Pearson correlation analysis demonstrated a positive linear relationship between the expression of γH2AX, pATM, Chk2, and p53, which was evident only in the ZEN group. This suggests that ZEN-induced damage may be associated with the activation of the γH2AX-pATM-Chk2-p53 signaling pathway (Table 4).

**Table 4:** Correlation values among parameters.

Variables	Pearson correlations			
	γH2AX	pATM	p53	Chk2
γH2AX	1.0000			
pATM	0.8062^***^	1.0000		
p53	0.8807^***^	0.9514^***^	1.0000	
Chk2	0.8450^***^	0.9678^***^	0.9604^***^	1.0000

The correlation matrix is symmetrical to the main diagonal, so it is sufficient to display the values of the lower triangle. ***Correlations are significant at *p* < 0.001.

### 3.4 Melatonin inhibits ZEN-induced cytotoxicity in GRM02 cells

**Figure 5:**
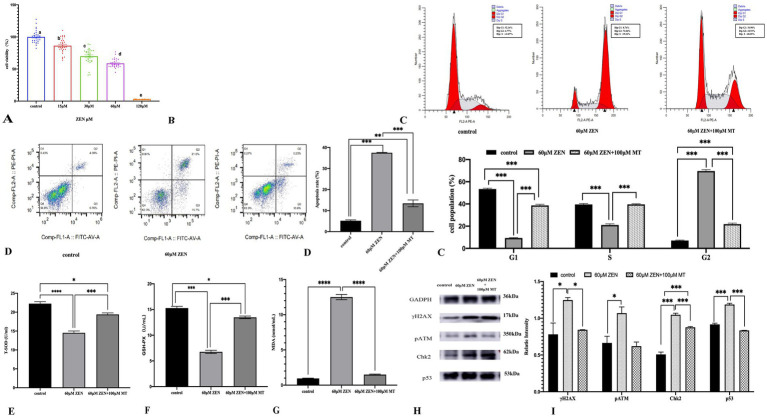
Various graphs and charts display the effects of different concentrations of ZEN on cell viability, apoptosis, cell cycle distribution, oxidative stress markers, and protein expression. Panel **(A)** shows a bar graph of cell viability percentages, decreasing with higher ZEN concentrations. Panel **(B)** presents flow cytometry histograms for cell cycle analysis under different conditions. Panel **(C)** includes bar charts comparing apoptosis rates and cell population percentages in various cell cycle phases. Panels **(D–G)** illustrate oxidative stress markers, while Panel **(H)** shows protein expression via Western blotting. Panel **(I)** displays a bar graph of relative protein intensities, highlighting statistical significance in changes.

Treatment with various concentrations of ZEN (0 μM, 15 μM, 30 μM, 60 μM, 120 μM) significantly reduced GRM02 cell viability in a dose-dependent manner, with statistical significance between groups (*p* < 0.05). The IC50 for ZEN was calculated to be 60.28 μM, setting the experimental concentration at 60 μM (Figure 5A). Combining 60 μM ZEN with different concentrations of MT (1 μM, 10 μM, 100 μM, 1,000 μM) for 24 h showed that MT (100 μM) significantly improved cell viability compared to ZEN alone (*p* < 0.05). However, MT (1,000 μM) reduced cell viability (*p* < 0.001), indicating toxicity at this concentration (Figure 5B). Thus, ZEN (60 μM) and MT (100 μM) were selected for subsequent experiments.

Cell cycle analysis revealed that ZEN (60 μM) treatment arrested GRM02 cells in the G2/M phase (69.62 ± 1.29% vs. control 7.00 ± 0.50%; *p* < 0.001). MT (100 μM) combined with ZEN (60 μM) maintained this arrest (21.75 ± 0.95% vs. control 7.00 ± 0.50%) and reduced the proportion of cells in G0/G1 and S phases (*p* < 0.001). Compared to ZEN alone, the combination increased G0/G1 and S phase cells (*p* < 0.001; Figure 5C). Apoptosis assays showed that ZEN (60 μM) increased apoptosis to 37.49 ± 0.15% (p < 0.001), while MT (100 μM) + ZEN (60 μM) reduced this to 13.44 ± 1.62% (*p* < 0.001; Figure 5D).

Oxidative stress markers indicated that ZEN (60 μM) reduced T-SOD and GSH-PX levels (*p* < 0.001) and increased MDA (*p* < 0.001). The MT (100 μM) + ZEN (60 μM) group showed increased T-SOD and GSH-PX levels compared to ZEN alone (*p* < 0.001), with no significant change in MDA levels (*p* > 0.05; Figures 5E–G).

DNA damage-related proteins showed that ZEN (60 μM) increased γH2AX, pATM, Chk2, and p53 (*p* < 0.05 to *p* < 0.001), while MT (100 μM) + ZEN (60 μM) reduced γH2AX, pATM, and p53 expression (*p* < 0.05), but increased Chk2 (*p* < 0.001; Figures 5H,I).

### 3.5 Melatonin ameliorates ZEN-induced DNA damage in mouse ovarian granulosa cells (GRM02) via the ATM-Chk2-p53 pathway

To further investigate the role of melatonin in alleviating ZEN-induced GRM02 cell damage through the reduction of oxidative stress-induced DNA damage, ATM knockout (ATM−/−) and ATM-activated (ATM-activator) GRM02 cells were generated. Six experimental groups were established to assess the effects of ZEN exposure and combined treatment with ZEN and melatonin (MT).

**Figure 6:**
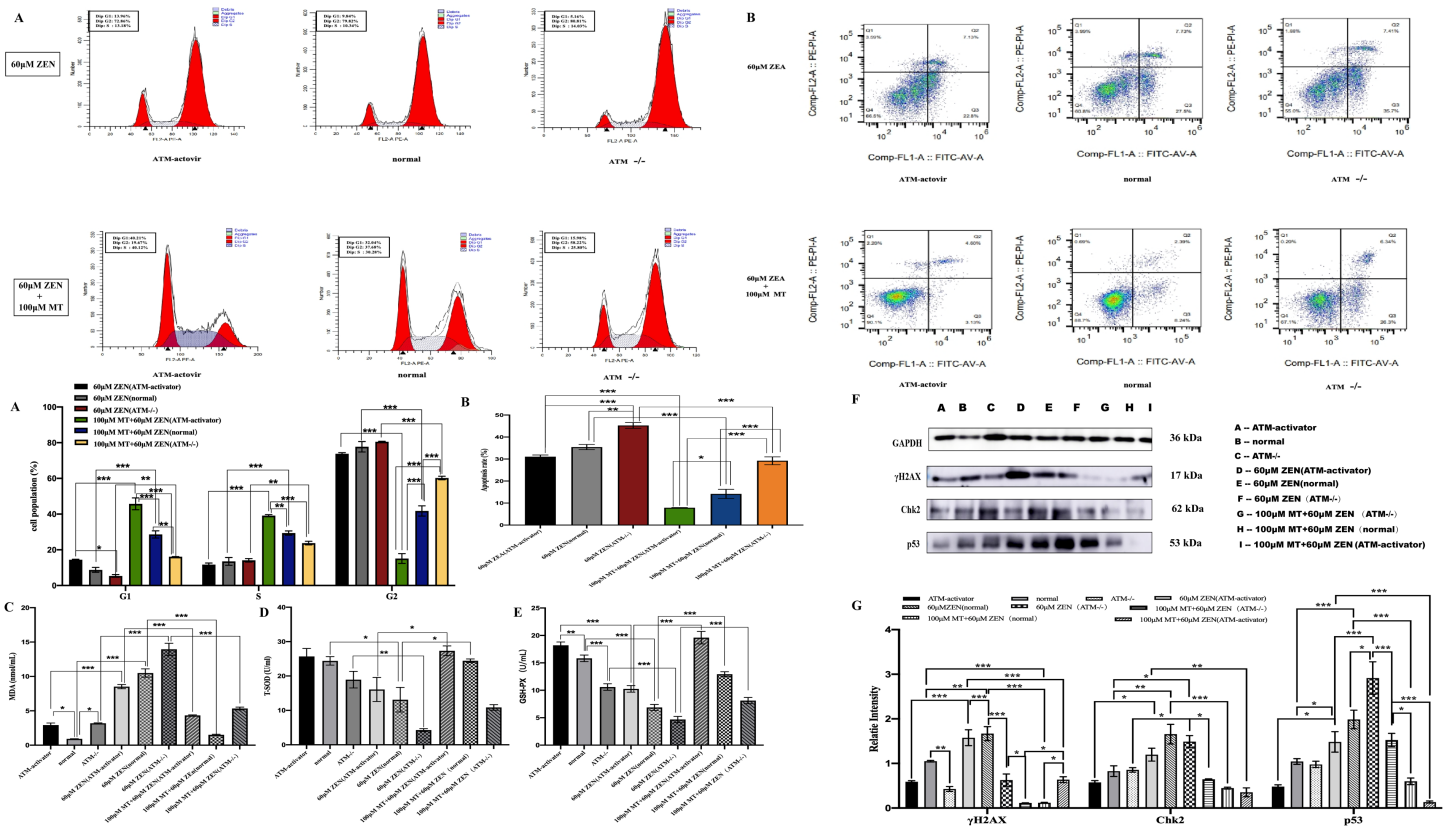
Melatonin attenuates cellular damage induced by ZEN by mediating the classical DNA damage pathway γH2AX-pATM-Chk2-P53. Panel **(A)** includes bar charts comparing cell population percentages in various cell cycle phases. Panel **(B)** provides bar charts comparing apoptosis rates. Panels **(C–E)** illustrate oxidative stress markers. Panel **(F)** shows protein expression via Western blotting. Panel **(G)** displays a bar graph of relative protein intensities, highlighting statistical significance in changes.

Cell cycle analysis revealed that treatment with ZEN (60 μM) predominantly caused G2/M phase arrest in all three groups (normal, ATM−/− and ATM-activator). The proportions of G2/M phase cells were 77.73 ± 2.94% in the normal group, 80.54 ± 0.26% in the ATM−/− group, and 73.81 ± 0.60% in the ATM-activator group, with no significant differences observed between the groups (*p* > 0.05). The proportion of cells arrested in the G0/G1 phase (normal 9.34 ± 0.43%, ATM−/− 5.34 ± 0.75%, ATM-activator 14.45 ± 0.27%) and the S phase (normal 21.05 ± 1.07%, ATM−/− 23.73 ± 1.04%, ATM-activator 39.15 ± 0.58%) showed slight differences, with a significantly higher proportion of G0/G1 phase arrest in the ATM-activator group compared to the ATM−/− group (*p* < 0.05). In response to combined ZEN (60 μM) and MT (100 μM) treatment, the ATM-activator group predominantly exhibited G0/G1 phase arrest, while the ATM−/− group remained in the G2/M phase. Significant differences were found in the proportions of G2/M phase cells: 41.78 ± 2.56% in the normal group, 60.19 ± 0.99% in the ATM−/− group, and 15.11 ± 2.73% in the ATM-activator group (*p* < 0.001). Proportions of cells arrested in G0/G1 were 28.63 ± 2.02% in the normal group, 16.08 ± 0.05% in the ATM−/− group, and 45.74 ± 3.31% in the ATM-activator group, with significant differences between the groups (*p* < 0.001). Similarly, the S phase proportions were 29.39 ± 1.18% in the normal group, 23.73 ± 1.04% in the ATM−/− group, and 39.15 ± 0.58% in the ATM-activator group, with significant differences between all pairs (*p* < 0.001). Comparison of cell cycle distributions before and after MT treatment showed that the proportions of cells in the G0/G1 and S phases significantly increased in all three groups (*p* < 0.001), while the proportions of G2/M phase cells significantly decreased (*p* < 0.001; Figure 6A).

Apoptosis assays indicated that ZEN (60 μM) treatment increased the apoptosis rate in the ATM−/− group (45.28 ± 1.32%) compared to the normal group (35.44 ± 1.19%; *p* < 0.01), while the ATM-activator group (31.07 ± 0.77%) showed a lower apoptosis rate, which was not significantly different from the control group (*p* > 0.05). After combined ZEN (60 μM) and MT (100 μM) treatment, the apoptosis rate in the ATM−/− group (29.23 ± 1.79%) remained significantly higher than in the normal group (14.17 ± 2.05%; *p* < 0.001), whereas the ATM-activator group (7.90 ± 0.09%) exhibited a significant reduction compared to the control group (*p* < 0.05). Apoptosis rates in the ATM−/− group were significantly higher than in the ATM-activator group (*p* < 0.001; Figure 6B).

Oxidative stress markers showed that ZEN (60 μM) treatment significantly decreased T-SOD and GSH-PX expression (*p* < 0.05) and increased MDA levels (*p* < 0.001) in all three groups. Compared to ZEN treatment alone, ZEN (60 μM) combined with MT (100 μM) treatment significantly increased T-SOD and GSH-PX expression (*p* < 0.001) and decreased MDA levels (*p* < 0.001; Figures 6C–E).

Western blot analysis of DNA damage-related proteins showed that, without treatment, the ATM−/− group had significantly lower γH2AX expression compared to the normal group (*p* < 0.05), while the ATM-activator group showed no significant difference (*p* > 0.05). Both the ATM−/− and ATM-activator groups had lower Chk2 and p53 expression compared to the normal group, but differences were not statistically significant (*p* > 0.05). After ZEN (60 μM) treatment, γH2AX expression was significantly decreased in the ATM−/− group compared to the normal group (*p* < 0.001), while expression in the ATM-activator group showed no significant change (*p* > 0.05). Chk2 expression increased in the ATM−/− group, while it decreased in the ATM-activator group, but differences were not significant (*p* > 0.05). p53 expression was significantly higher in the ATM−/− group (*p* < 0.001), while the ATM-activator group showed decreased expression (*p* > 0.05). After ZEN (60 μM) treatment, γH2AX expression increased significantly in the normal group (*p* < 0.01), but the increase was not significant in the ATM−/− group (*p* > 0.05). In the ATM-activator group, γH2AX expression increased significantly (*p* < 0.001). Similarly, Chk2 expression increased in all groups (*p* < 0.05), and p53 expression increased significantly in the normal and ATM−/− groups (*p* < 0.05 and *p* < 0.001, respectively).

After combined ZEN (60 μM) and MT (100 μM) treatment, γH2AX expression was significantly reduced in all three groups (*p* < 0.001), with the ATM−/− group showing a significant reduction (*p* < 0.05), and the ATM-activator group showing a more marked reduction (*p* < 0.001). Chk2 expression decreased significantly in the normal group (*p* < 0.001), with both the ATM−/− and ATM-activator groups showing significant decreases (*p* < 0.01). Similarly, p53 expression decreased significantly in all three groups (*p* < 0.001; Figures 6F,G).

## 4 Discussion

Zearalenone (ZEN), a mycotoxin produced by Fusarium species, is commonly found in improperly stored grains and their derivatives, posing a significant threat to food safety (30). ZEN and its metabolites exhibit non-steroidal estrogen-like activity by competing with estrogen receptors. Chronic excessive exposure to ZEN can result in reproductive disorders, oxidative stress, apoptosis, and various toxic effects. Our study demonstrates that exposure to 0.8 mg/kg ZEN results in a 21.3% decrease in ovarian weight, disruption of the estrous cycle (with the absence of the estrus phase), loss of mature follicles, a 61.3% reduction in the number of primordial follicles (compared to the control group), an increase in atretic follicles, disordered arrangement of granulosa cells, reduced follicular fluid content, ovarian fibrosis, and decreased serum levels of E2, FSH, and LH. These findings are consistent with those of Minervini et al. (31), who also reported that ZEN inhibits the secretion of FSH, preventing follicular maturation during the pre-ovulatory phase and impairing ovulation. Empirical research has demonstrated that even low concentrations of ZEN (250 μg/kg and 750 μg/kg) along with its metabolites, can significantly influence the estrogen and progesterone levels in laying hens. This, in turn, affects their physiological functions as well as the quantity and quality of eggs produced (32, 33). Additionally, other studies have shown that ZEN significantly damages the morphology of primordial and primary follicles in cultured sheep ovarian tissues, leading to premature follicular atresia and an increased number of atretic follicles, thereby disrupting normal follicular development and quantity (34). Research indicates that the concentrations of ZEN and its metabolites in porcine follicular fluid are notably elevated, measuring approximately 38.9 pg./mL for *α*-ZEL and 17.6 pg./mL for *β*-ZEL. These elevated concentrations within the follicles may have a direct impact on follicle development and ovulation (35). Furthermore, an investigation into the potential effects of fungal toxins in human follicular fluid on reproductive outcomes, conducted on 25 female patients undergoing *in vitro* fertilization (IVF) treatment, revealed that the levels of ZEN and its metabolites were significantly higher in follicular fluid compared to serum (mean ± SD for follicular fluid Zearalenone: 241.93 ± 179.34 pg./mL vs. serum Zearalenone: 81.34 ± 100.58 pg./mL; follicular fluid alpha-Zearalenol: 554.56 ± 194.18 pg./mL vs. serum alpha-Zearalenol: 192.52 ± 121.72 pg./mL). Additionally, a positive correlation was observed between ZEN and estradiol (E2) levels. The study concluded that the association between mycotoxins and measurable hormone levels (E2, P4) in follicular fluid suggests that mycotoxins may affect follicle maturation and hormone synthesis (36).

Melatonin (MT), an indoleamine primarily synthesized and secreted by the pineal gland, plays a crucial role in regulating circadian rhythms, behavior, immune responses, and reproductive functions in mammals (37). Our study further observed that co-treatment with MT at different concentrations significantly alleviates the damage caused by ZEN to mouse ovarian function. Specifically, 40 mg/kg MT significantly reversed the toxicity induced by ZEN, restoring ovarian weight to 92.4% of the control group (*p* < 0.05), stabilizing the estrous cycle, promoting follicular development and maturation, improving the orderly arrangement of granulosa cells, enriching follicular fluid, and reducing the occurrence of atretic follicles. Moreover, MT exhibited a dose-dependent protective effect: in the recovery of primordial follicle count. This effect may be related to the dose-dependent accumulation of MT’s antioxidant capacity. Furthermore, 40 mg/kg MT significantly increased serum levels of E2, FSH, and LH in female mice. These results suggest that MT regulates the hypothalamic–pituitary-gonadal (HPG) axis by modulating GnRH expression, which in turn controls the secretion of FSH and LH, thus promoting follicular development (38). Similar studies have found that melatonin implantation in sheep and goats significantly reduces the atresia rate of medium and large follicles, increasing the number and quality of dominant follicles and accelerating follicular growth, indicating the protective role of MT on ovarian function (39). Despite the absence of universally accepted clinical diagnostic criteria for assessing melatonin concentrations in the blood and follicular fluid of healthy adults, research has demonstrated that melatonin levels in follicular fluid generally range from 30 to 200 pg./mL, which is significantly higher than the concentrations found in blood (40, 41). This phenomenon was initially reported by Brzezinski in 1987, who observed a nocturnal peak serum melatonin concentration of 80–120 pg./mL and a follicular fluid level of 36.5 ± 4.8 pg./mL in a study involving 32 healthy women (42).

The toxicity of ZEN and its metabolites is not only estrogenic but is also linked to the induction of oxidative stress (5). Oxidative stress refers to an imbalance between the production of reactive oxygen species (ROS) and reactive nitrogen species (RNS) and the body’s antioxidant defense mechanisms, leading to tissue damage (43). In this study, we observed that ZEN exposure led to significant reductions in serum T-AOC, T-SOD, and GSH-PX levels, accompanied by an increase in MDA levels, indicating that ZEN induces oxidative stress and impairs antioxidant capacity in the body. MT, as an endogenous antioxidant, can scavenge free radicals and enhance resistance to oxidative stress, thereby preventing cellular damage. Additionally, MT not only acts as a direct free radical scavenger but also indirectly promotes the expression and activation of antioxidant enzymes such as SOD and GSH-PX and inhibits the expression of NOS (31, 44). After co-treatment with different concentrations of MT, we found that the reduction in serum T-AOC, T-SOD, and GSH-PX levels was less pronounced, and MDA levels did not significantly increase in the ZEN (0.8 mg/kg) + MT (40 mg/kg) group, indicating that MT mitigated the oxidative stress induced by ZEN.

Our study also demonstrated that ZEN exposure increased the expression of DNA damage-related proteins (γH2AX, pATM, Chk2, and p53) in mouse ovarian tissues, suggesting that ZEN induces DNA damage during ovarian tissue damage. These findings are consistent with other studies that report that ZEN induces DNA breaks, disrupts DNA replication, and leads to apoptosis. ZEN exposure increases the expression of γH2AX, a DNA damage marker, and activates ATM (ataxia telangiectasia mutated), a key protein kinase in the DNA damage response. ATM phosphorylates downstream proteins like Chk2 and p53, initiating cell cycle checkpoints and DNA repair mechanisms (45). Furthermore, our experimental results showed that co-treatment with MT significantly reduced the expression of γH2AX, pATM, Chk2, and p53 in ovarian tissues, suggesting that MT effectively mitigates ZEN-induced DNA damage.

ZEN also induces cell cycle arrest through oxidative stress. In our GRM02 cell model, we observed that ZEN (60 μM) significantly induced G2/M phase arrest, further validating ZEN’s impact on the cell cycle (46, 47). The DNA damage caused by ZEN activates ATM, leading to the phosphorylation of Chk2 and p53, which in turn activates cell cycle checkpoints (48, 49). These findings were confirmed by the increased expression of γH2AX, pATM, Chk2, and p53, indicating that ZEN induces DNA damage through oxidative stress and leads to G2/M phase cell cycle arrest.

With MT intervention, the G2/M phase arrest was reduced, and the cell cycle progressed normally. This may be attributed to MT’s regulation of cell cycle-related proteins (such as p53 and Chk2), helping restore normal cell cycle progression by enhancing antioxidant enzyme activity and reducing ROS levels. The results showed that MT decreased the expression of DNA damage-related proteins, further supporting the conclusion that MT mitigates ZEN-induced oxidative stress and DNA damage.

ATM (Ataxia Telangiectasia Mutated) protein is a serine/threonine protein kinase, and its deficiency is closely related to the human neurodegenerative disease Ataxia-Telangiectasia (AT) (50). As a key regulator in the signal transduction of DNA double-strand breaks (DSBs), ATM plays a pivotal role in DNA damage response (DDR) (50). ATM exerts its function by sensing DSBs, activating cell cycle checkpoints, promoting cell cycle arrest, facilitating DNA repair, and inducing apoptosis in the case of severe damage to prevent the proliferation of damaged cells (51). Thus, ATM plays a crucial role in maintaining cell health and genomic stability. Chk2 is an important protein kinase involved in the regulation of cell cycle checkpoints, especially in DNA damage response and repair processes. ATM accumulates at the site of DNA damage and phosphorylates Chk2. The activated Chk2 phosphorylates various proteins, such as p53, to enhance their activity in DNA damage response, leading to cell cycle arrest or apoptosis. When DNA damage is severe, p53 initiates apoptosis to prevent the growth of mutated cells (52, 53). The ATM-Chk2-p53 signaling pathway is a critical pathway for cells to respond to DNA damage, playing a vital role in maintaining genomic integrity and regulating apoptosis (54).

The activation of ATM depends on multiple signaling pathways, including responses to oxidative stress. Studies have shown that ATM can be activated by oxidative stress, highlighting its important role in regulating cellular ROS levels (55). In this study, ATM knockout (ATM−/−) GRM02 cells and ATM activator (ATM-activator) GRM02 cells were constructed to investigate the role of the ATM-Chk2-p53 signaling pathway in ZEN-induced DNA damage and to explore the mechanism by which melatonin (MT) intervenes in DNA damage through this pathway.

In the results of oxidative stress indicators, the ATM−/− group showed a decrease in T-SOD expression, but the difference was not significant, while GSH-PX decreased significantly, and MDA expression increased compared to the normal group. These results suggest that ATM knockout affected the cell’s antioxidant capacity. After ZEN induction, the antioxidant capacity (T-SOD, GSH-PX) decreased, and MDA expression increased in the ATM−/− group compared to the normal group. This suggests that the absence of ATM increased the sensitivity of the cells to oxidative stress, making them more susceptible to oxidative damage. Similar studies have also concluded that cells lacking ATM exhibit hypersensitivity to oxidative stress, except for DSBs (56). After co-treatment with ZEN and MT, the antioxidant capacity in the ATM−/− group was the weakest (T-SOD increased only from 4.27 to 10.86), with no significant difference compared to the normal group, further confirming that ATM is a key mediator in the antioxidant effects of MT.

The cell cycle results from the ATM−/− group indicated that ZEN treatment caused more cell cycle arrest at the G2/M phase. After co-treatment with ZEN and MT, the cell cycle improved but still predominantly remained in the G2/M phase compared to the ZEN-only group. This suggests that the absence of ATM prevents effective activation of early checkpoints, and the cells can only rely on G2/M phase arrest to prevent damaged DNA from entering mitosis, thus preventing genomic instability and potential cell death (10). Research has found that the ATR pathway can function in ATM-deficient cells by regulating the G2/M checkpoint to prevent damaged DNA from entering mitosis (57). This mechanism indicates that although ATM is crucial for DNA damage response, cells can partially compensate for the impact of ATM deficiency through other pathways.

Cell apoptosis experiments in the ATM−/− group showed that ZEN alone significantly increased apoptosis rates compared to the normal and ATM-activator groups. This observation suggests a dual role of ATM in DNA damage response: 1. Normal ATM activity can reduce apoptosis by repairing DSBs or activating cell cycle arrest. In the ATM−/− group, due to the lack of repair ability, damage accumulates, triggering a stronger apoptosis signal. 2. Under extreme damage, ATM might also accelerate apoptosis by phosphorylating p53 or pro-apoptotic proteins such as Bax. After co-treatment with MT, apoptosis rates in all three groups significantly decreased, but the ATM−/− group still exhibited higher apoptosis than the other two groups, indicating that MT’s anti-apoptotic effect is partially dependent on ATM activity. MT likely reduces ROS-induced DNA damage and lowers the need for ATM pathway activation by restoring T-SOD and GSH-PX activity.

The expression of DNA damage-related proteins in the ATM−/− group indicated that, without any drug intervention, the γH2AX expression level was significantly lower than in the normal group. This result may be due to ATM deficiency, leading to delayed and less efficient phosphorylation of H2AX. ATM is one of the major kinases for γH2AX formation, and the lack of ATM directly affects the generation of γH2AX (58). The expression levels of Chk2 and p53 showed no significant differences compared to the normal group, suggesting that ATM deficiency has a weaker regulatory effect on these proteins in resting cells, likely because DNA damage signals are less frequent in resting cells, reducing the need for ATM activation. This result is consistent with similar findings in studies of macrophages and T cells by Dhariwala et al. (59). After ZEN treatment, γH2AX, Chk2, and p53 increased in all three groups, but the ATM−/− group showed the smallest increase in γH2AX (*p* > 0.05), further confirming that ATM is the primary kinase for γH2AX phosphorylation following ZEN-induced DSBs. Furthermore, the p53 level in the ATM−/− group increased significantly (*p* < 0.001), which may be the result of compensatory activation of the ATR or DNA-PKcs pathways. Some studies have indicated that ZEN can activate the ATR pathway, triggering a series of phosphorylation events, including Chk2, and is related to oxidative stress, although its effect is less efficient than ATM (60, 61)^.^ This explains why DNA damage-related protein expression increases in ATM knockout cells after ZEN exposure. Other studies suggest that ATM deficiency impairs DNA damage response mechanisms but also increases cell susceptibility to apoptosis, which is especially evident in cancer cells (62).

After co-treatment with MT, DNA damage-related proteins significantly decreased in all three groups, but the decrease in γH2AX and Chk2 in the ATM−/− group was the smallest, indicating that MT promotes repair mainly through the ATM-dependent pathway: 1. Direct activation of ATM: MT may enhance ATM autophosphorylation by modulating the redox state of cells. 2. Co-activation of downstream factors: MT may upregulate the expression of BRCA1 or Rad51, accelerating homologous recombination repair (HRR). Some studies suggest that MT can directly induce phosphorylation of p53, inhibiting cell proliferation in tumors and preventing DNA damage accumulation (63). On the other hand, ATM knockout partially compromised MT’s ability to mitigate DNA damage, which aligns with other findings that “MT has been shown to improve the efficiency of DNA repair mechanisms in cells affected by oxidative stress” (64).

In the ATM-activator group, the oxidative stress results showed that, without treatment, the baseline T-SOD and GSH-PX activities were significantly higher than in the normal group (*p* < 0.05), while the MDA level was significantly elevated. This seemingly contradictory phenomenon may be due to the continuous activation of ATM, triggering Nrf2 nuclear translocation (even without stress), driving the transcription of SOD1/2 and GPX1 to cope with basal oxidative stress (55, 56). The ATM-activator group may increase membrane fluidity by upregulating fatty acid desaturases (such as SCD1), while sacrificing some lipid peroxidation products (e.g., MDA) to maintain cell function (60, 65). This is linked to the ability of ATM to be activated directly by oxidation, which regulates intracellular ROS levels (11). After ZEN exposure, although T-SOD and GSH-PX activities in the ATM-activator group significantly decreased (compared to before treatment), their absolute levels were still higher than in the other groups. After co-treatment with MT, T-SOD recovered to 27.32 (vs. normal group 23.44, *p* < 0.05) and GSH-PX to 19.60 (vs. normal group 12.93, *p* < 0.001). This suggests a synergistic antioxidant effect between ATM and MT, with MT potentially enhancing Nrf2 transcriptional activity (in parallel with ATM signaling) to further boost the expression of antioxidant enzymes (66).

Cell cycle results from the ATM-activator group showed that under ZEN treatment, although the ATM-activator group still had a majority of cells arrested in the G2/M phase, the proportion of cells in the G0/G1 phase was significantly higher than in the ATM−/− group. This suggests that excessive activation of ATM may partially restore G1/S checkpoint functionality. ATM phosphorylates p53 (Ser15), enhancing its stability, and subsequently upregulates p21 expression, inhibiting CDK2/cyclin E activity and arresting cells in the G1 phase (67). After co-treatment with MT, the proportion of cells in the G0/G1 phase in the ATM-activator group rose to 45.74%, significantly higher than in the other groups, with the highest proportion of cells arrested in S phase. This further supports the idea that ATM activation enhances the sensitivity of cells to S phase damage. Moreover, the ATM-activator group may inhibit CDC25A phosphatase (with increased expression of Chk2), a key regulator of cell cycle progression, by activating Chk2. CDC25A promotes cell cycle progression by dephosphorylating CDK (Cyclin-Dependent Kinase), but under DNA damage, CDC25A activity needs to be inhibited to prevent damaged DNA from entering S phase for replication. The ATM-CHK2 pathway maintains the inhibitory phosphorylation state of CDK2 (Tyr15) by inhibiting CDC25A, thereby reinforcing G1/S arrest and preventing cells from entering S phase before DNA repair is complete (68). MT may block CDK2/cyclin A activity (in collaboration with ATM) and arrest cells at the G1/S transition rather than the classical S phase checkpoint.

In the ATM-activator group, apoptosis experiments showed that although the baseline apoptosis rate (31.07%) was not significantly different from the normal group (35.44%), after co-treatment with MT, the apoptosis rate decreased to 7.90%, significantly lower than the other groups. This indicates that excessive activation of ATM enhances DNA repair capacity and reduces apoptosis signals. In our study, the γH2AX level in the ATM-activator group decreased the most after co-treatment with MT, suggesting that excessive ATM activation promotes homologous recombination repair (HRR), efficiently removing DNA damage and reducing apoptosis signals triggered by persistent damage (69). Additionally, MT may inhibit the pro-apoptotic function of p53 through SIRT1-mediated deacetylation (70). It is noteworthy that, under ZEN treatment alone, the apoptosis rate in the ATM-activator group was lower than in the normal group, suggesting that excessive activation of ATM could repair ZEN-induced DNA damage, reducing leakage of apoptosis signals.

In the ATM-activator group, DNA damage-related protein expression showed that, without treatment, γH2AX levels were significantly lower than in the normal group. This suggests that mild activation of ATM in resting cells can pre-activate some DNA repair factors, such as key enzymes of non-homologous end joining (NHEJ) or early involvement in homologous recombination repair (HRR). These pre-activated repair factors help cells respond quickly to DNA damage, thereby reducing the expression level of γH2AX caused by repair failure or delays (71). After ZEN treatment, the ATM-activator group showed the greatest increase in γH2AX, Chk2, and p53, with the most significant increase in γH2AX expression, suggesting that ATM activation accelerates the recognition of DSBs by the MRN complex (MRE11-RAD50-NBS1), and amplifies ATM kinase activity through autophosphorylation (Ser1981) (72, 73). ATM activation may promote H2AX phosphorylation over a larger region surrounding the damage site, forming more prominent γH2AX foci (74). Chk2 expression was significantly elevated, indicating that ATM is the primary driver of Chk2 phosphorylation after ZEN-induced DNA damage, and that the compensatory effects of ATR or DNA-PKcs are limited. Excessive activation of ATM likely expands Chk2’s substrate range (e.g., phosphorylating CDC25A Ser124), enhancing the strength of G1/S arrest. After MT co-treatment, γH2AX and Chk2 decreased the most in the ATM-activator group, indicating that MT promoted efficient repair through HRR and NHEJ, reducing DNA damage marker accumulation and decreasing Chk2 phosphorylation levels.

Our study acknowledges certain limitations. Primarily, the quantification of ZEA and MT in blood and follicular fluid represents a critical component that is currently under investigation in our ongoing research efforts. We are in the process of refining our methodological approaches and intend to integrate these advancements into subsequent experimental frameworks. Additionally, while this study concentrated on elucidating the role of the ATM-Chk2-p53 signaling pathway in ZEA-induced oxidative damage, it is important to recognize the complexity of oxidative stress-related signaling networks. Other pathways, such as Nrf2-ARE and MAPK, may also significantly contribute to these processes. Future research should consider examining whether the protective effects of MT are mediated through alternative signaling mechanisms.

## 5 Conclusion

This study determined that ZEN induces oxidative stress in mouse ovarian tissue and GRM02 cells, resulting in functional impairment of the ovarian tissue and oxidative damage in the GRM02 cells. MT was found to alleviate oxidative stress, enhance the functionality of mouse ovarian tissue, and mitigate oxidative damage in GRM02 cells. The ATM-Chk2-p53 signaling pathway was identified as a pivotal mechanism in MT’s attenuation of ZEN-induced oxidative damage in GRM02 cells. This research elucidates the mechanism underlying ZEN’s reproductive toxicity and provides a theoretical foundation for MT’s protective effects, while also offering experimental evidence supporting MT’s potential as a protective feed additive.

## Data Availability

The original contributions presented in the study are included in the article/supplementary material, further inquiries can be directed to the corresponding author.
